# Embryonic Origins of Virus-Induced Hearing Loss: Overview of Molecular Etiology

**DOI:** 10.3390/v13010071

**Published:** 2021-01-06

**Authors:** Maryam Karimi-Boroujeni, Ali Zahedi-Amiri, Kevin M. Coombs

**Affiliations:** 1 School of Rehabilitation Sciences, Faculty of Health Sciences, University of Ottawa, Ottawa, ON K1H 8M5, Canada; mkari052@uottawa.ca; 2 Department of Medical Microbiology and Infectious Diseases, University of Manitoba, Winnipeg, MB R3E 0J9, Canada; zahediaa@myumanitoba.ca; 3 Manitoba Centre for Proteomics and Systems Biology, Winnipeg, MB R3E 3P4, Canada; 4 Children’s Hospital Research Institute of Manitoba, University of Manitoba, Winnipeg, MB R3E 3P4, Canada

**Keywords:** hearing loss, viruses, embryogenesis, inner ear formation, auditory system development

## Abstract

Hearing loss, one of the most prevalent chronic health conditions, affects around half a billion people worldwide, including 34 million children. The World Health Organization estimates that the prevalence of disabling hearing loss will increase to over 900 million people by 2050. Many cases of congenital hearing loss are triggered by viral infections during different stages of pregnancy. However, the molecular mechanisms by which viruses induce hearing loss are not sufficiently explored, especially cases that are of embryonic origins. The present review first describes the cellular and molecular characteristics of the auditory system development at early stages of embryogenesis. These developmental hallmarks, which initiate upon axial specification of the otic placode as the primary root of the inner ear morphogenesis, involve the stage-specific regulation of several molecules and pathways, such as retinoic acid signaling, Sonic hedgehog, and Wnt. Different RNA and DNA viruses contributing to congenital and acquired hearing loss are then discussed in terms of their potential effects on the expression of molecules that control the formation of the auditory and vestibular compartments following otic vesicle differentiation. Among these viruses, cytomegalovirus and herpes simplex virus appear to have the most effect upon initial molecular determinants of inner ear development. Moreover, of the molecules governing the inner ear development at initial stages, SOX2, FGFR3, and CDKN1B are more affected by viruses causing either congenital or acquired hearing loss. Abnormalities in the function or expression of these molecules influence processes like cochlear development and production of inner ear hair and supporting cells. Nevertheless, because most of such virus–host interactions were studied in unrelated tissues, further validations are needed to confirm whether these viruses can mediate the same effects in physiologically relevant models simulating otic vesicle specification and growth.

## 1. Introduction

Prelingual hearing loss is present before the normal development of speech and language. This condition emerges at birth in 3 to 6 out of every 1000 neonates and is audiometrically classified based on its intensity, ranging from slight to profound and total deafness [1,2]. Conductive hearing loss (CHL) and sensorineural hearing loss (SNHL) are the two main types of hearing loss, which root in defective sound wave transfer and abnormality in inner ear or auditory nerve, respectively. Generally, prelingual hearing loss includes all types of congenital hearing impairments and are mainly inherited through non-syndromic autosomal recessive disorders that usually involve mutations in genes regulating sensory perception and mechanoreception of sound waves [3].

Apart from genetic factors, many cases of congenital hearing loss with acquired and environmental origins are caused by viral infections during pregnancy. The cytomegalovirus (CMV) is the most common example of these viral pathogens that causes deafness in newborns and progressive SNHL in children. About 50% of CMV congenital infections are believed to happen following initial maternal exposure and the subsequent transplacental passage of this virus into fetal tissues [4,5]. Due to latency and reactivation capacities of this virus, most cases of congenital CMV infection might manifest asymptomatically up to years after birth and often lead to bilateral SNHL in 15% of individuals who remained undiagnosed at their neonatal stages [6,7,8]. Conversely, almost half of infants with symptomatic congenital CMV infection have been observed to develop hearing loss, in addition to several other permanent damages such as seizures, cerebral palsy, and visual defects [5,6]. It is not clear whether the CMV-triggered hearing loss is directly caused by viral components or host immune response to inner ear infection. Both clinical and in vivo studies have detected inflammation of cochlea after CMV infection and the presence of viral antigens within the structures of inner ear [8,9]. Similarly, congenital Rubella virus (RV) infection leads to bilateral SNHL through damaging the cochlea among nearly 60% of infected offspring; however, this harm was demonstrated to be conducted by virus-induced apoptosis [10,11]. Moreover, the effect of RV on strial tissues has been reported to contribute to structural changes in endolymph [12]. Accordingly, the RV-mediated hearing loss might originate directly from cytopathological effects that this virus establishes in components of the internal ear rather than from inflammatory signals per se. Congenital RV infection is usually transmitted to the developing fetus during the first trimester of pregnancy, a stage in which the fetal tissues are vulnerable and not yet fully immunocompetent against pathogens [12]. It seems likely that the vast majority of SNHL cases following RV infection are due to the direct interreferences of this virus with tissues forming the fetal auditory system. Such virus-specific pathogenic differences suggest that the teratogen viruses like CMV and RV could utilize entirely different cellular and molecular mechanisms to cause hearing loss either at developmental or neonatal stages. These etiological differences also could be seen in some cases occurring after infection with viruses that are known to cause both congenital and acquired haring loss, such as human immunodeficiency virus (HIV) and herpes simplex virus (HSV). For instance, although HIV particles were seen to infect auditory and vestibular hair cells, studies have indicated abnormalities in auditory brainstem responses (ABRs) in patients who developed SNHL and/or CHL following this infection, which suggest the auditory nerve might be targeted by HIV as well [13,14,15]. Further, human and animal investigations on HSV1/2-related unilateral or bilateral SNHL have not only discovered fibrosis and atrophy within different parts of the vestibulocochlear organ but also have found viral proteins in afferent and efferent nerve fibers innervating the cochlea [11,16]. This highlights the fact that the viral origins of hearing loss in newborns could vary in their pathogenesis, whether the virus is known to be teratogenic or not.

Despite the development of vaccines and antivirals, the treatment of virus-induced hearing loss faces several obstacles, probably because of different biofunctions and tropisms that each virus exploits that result in hearing loss. Furthermore, temporary or permanent molecular effects of many other viruses on the auditory system have remained elusive both in adults and infants. Some viruses rarely manifest as obvious congenital infections but might be capable of disturbing the embryonic or fetal development if they can cross the placental barrier during early pregnancy. Nevertheless, the exact mechanisms by which viruses cause hearing loss are not studied sufficiently at molecular level. Determining commonalities and differences in such molecular mechanisms helps to develop better therapies against viruses triggering hearing loss. The present review is oriented toward the prediction of potential molecular alterations that various viruses might induce in cells that develop into different parts of the inner ear and the auditory system during embryogenesis.

## 2. Initial Cellular Hallmarks of Auditory System Development

The embryonic development of the inner ear implicates complex cellular and molecular specifications in which any type of abnormality could result in hearing malfunction or impairment in offspring. During the third week of embryogenesis, cells that are situated on the outer surface of the ectoderm thicken to form the otic placode, an initial root of inner ear development through which vestibular and auditory systems shape up [17,18]. Subsequently, otic placode invaginates into the mesenchymal side of the hindbrain to create an auditory pit that detaches from neuronal ectoderm and converts into an enclosed otic vesicle (otocyst) [19,20]. This epithelial tissue receives crucial signaling information from neighboring cells to obtain anterior–posterior, dorsal–ventral, and medial–lateral axes [21]. The otic vesicle gradually differentiates into the membranous labyrinth after the fourth week of embryogenesis. Basically, the ventral wall of the otocyst contributes to the formation of the vestibulocochlear nerve and the endolymphatic sac. Moreover, via elongation and coiling upon itself, the ventral projection of the otic vesicle organizes the cochlear duct, a cavity that perceives sound and encompasses the organ of Corti [22]. The central region of otocyst develops into membranous vestibule, which includes saccule, utricle, and semicircular canals and involves balance regulation. Generally, three primitive types of cell fate determinations are assumed to be present at the early stage of otocyst specification, including sensory, non-sensory, and neural cell commitments [23]. An epithelial layer of otic vesicle contains cells tending to differentiate into neuronal populations within the vestibulocochlear ganglion. Sensory hair cells and supporting cells derive from the cells with sensory fate. The non-sensory cells generate cells constituting structural parts of the inner ear like semicircular canals and endolymphatic duct. All these post-otocyst differentiation events require tissue-specific and transient regulation of several signaling pathways and their member molecules at certain developmental stages.

## 3. Molecular Determinants of Inner Ear Development

Axial specification of the otic vesicle is one the earliest stage of inner ear development that progresses over time to regulate cell fate determinations. It initiates with changes in the anterior–posterior axis through acquiring signals from surrounding germ layers, eventually evolving into sensory tissues. According to in vivo studies, in addition to transcription factors *SOX2*, *SIX1*, and *NGN1*, other molecules like *FGF10* and *LFNG* are more highly expressed in the anterior part of the otocyst than in its posterior region [24,25,26,27]. This anterior axis is known as the neural sensory competent domain and contains *NGN1*^+^ cells that finally turn into neural progenitors migrating toward vestibulocochlear ganglion [28]. Non-sensory tissues originate from the posterior part. Controlling anterior–posterior patterning of otocyst also entails the regulation of retinoic acid signaling via its associated interacting molecules, such as *RALDH2*, *CYP26*, *TBX1*, *FGF8*, and *SOX3* [29,30,31,32]. Specification of dorsal–ventral axis relies on ectodermal signals from hindbrain, which comprise activation or inhibition functions of regulatory molecules within signaling pathways like Sonic hedgehog (SHh) and Wnt. For example, the distal part of the cochlear duct is dependent on high levels of SHh activators *GLI2* and *GLI3* to develop from the ventral region of the otocyst, while the formation of other structures including saccule, utricle, and proximal part of the cochlear duct, requires lower activity of SHh pathway for reducing the inhibitory function of *GLI3* repressor molecule [28,33,34]. Furthermore, regardless of primary hindbrain-derived Wnt signals that stimulate placode specification, the otic placode imparts *WNT1* and *WNT3A* expressions from the dorsal neural tube in order to conduct otic dorsal–ventral patterning [35,36]. Insufficient experimental evidence is available on how exactly the medial–lateral axis of otic vesicle becomes specified. However, it has been speculated that the expression of transcription factors *GBX2* and *PAX2* within the epithelial layers of otocyst might give directions to external signals from hindbrain that seem to be essential for medial–lateral specification [28,37]. This axial specification might be involved in the delamination of vestibular and auditory neuroblasts from the neural sensory domain. Fate-mapping studies on avian and murine models have shown that vestibular and auditory neuroblasts arise from lateral and medial areas of the neural sensory domain, respectively [38,39]. The transcription factor *LMX1A* is highly expressed in auditory neuroblasts, whereas vestibular neuroblasts produce considerable amounts of *FGF3* [28,40]. Variations in the type and function of molecules governing the otocyst axial specifications suggest that different signaling pathways might be altered to some extent at certain developmental stages to induce cell fate determination from the otic placode toward the generation of inner ear structures.

Some genes that play roles in axial specification can also regulate the determination of neural and sensory cell fates. For example, *NGN1*, which induces neurogenic cell fate among precursor populations shifting from neural sensory domain into vestibulocochlear ganglion, also was found expressed in sensory parts of the utricle and saccule, highlighting the proneural function of this molecule in creating both sensory and neuronal bases of inner ear development [41]. The establishment of neural sensory domain from the anterior region of otocyst itself needs *EYA1* phosphatase activity [28,42]. Furthermore, molecules controlling Notch signaling such as *RBPJ* and *JAG1* have been suggested to be crucial in sensory fate specification as their absence could mediate substantial sensory abnormalities [43,44,45]. Likewise, irrespective of a few specific genes and linked pathways, differentiation of non-sensory progenitors in the inner ear involves the activity of some factors that could mutually direct axial specification of the otic vesicle. External Wnt signals like Wnt1/Wnt3a, which originate from the dorsal region of the hindbrain, are one of these examples that control both dorsal–ventral patterning of otocyst and development of semicircular canals [46]. More specific molecules provoking the generation of these canals include but are not limited to homeobox transcription factors *DLX5* and *HMX3*, which interact with Wnt and FGF pathways, correspondingly [47,48,49,50,51]. Additionally, SHh signals, apart from their involvement in axial specification and forming the cochlear duct, shape the axes of semicircular canals [28,33]. Animal studies have demonstrated that the generation of these canals also could be triggered by the regulation of some genes that are expressed by surrounding mesenchymal tissues, such as *POU3F4* and *PRX* [52,53,54]. Moreover, together with SHh mediators and *TBX1*, *POU3F4* adjusts the outgrowth and proper coiling of the cochlear duct [28]. The compartments of cochlear duct, like organ of Corti, the stria vascularis, and Reissner’s membrane, are known to be localized under the influence of *JAG1*, *LFNG*, *SOX2*, *CDKN1B*, and *BMP4* [28,55,56]. Formation of sensory hair cells residing in the organ of Corti implicates transcription factor *ATOH1*, whose expression will be mostly limited to hair cells over time [57,58]. Sensory hair cells convert sound vibrations into electrical signals that can be received in auditory brainstem and cortex after amplification through auditory nerve. These cells are surrounded by supporting cells acting like glia. Generation of different supporting cells appear to be induced by signals from hair cells, which mainly consist of alterations in expression of *FGFR3*, *FGF8*, SPRY2, and *HEY2* [59,60,61,62,63]. Depending on the stage of embryogenesis, it is likely that any dysregulation in molecules and signaling pathways shaping the initial structures of inner ear could potentially lead to hearing impairments in the developing embryo.

## 4. Potential Molecular Origins of Virus-Induced Hearing Loss

Different RNA and DNA viruses tend to dysregulate intracellular molecules and signaling pathways, regardless of their tissue tropism and proven teratogenicity. Because they are capable of replicating within post-otocyst differentiation-derived cells and organs, viruses could be considered one of the potential reasons for hearing loss that might evolve following virus-triggered abnormalities in inner ear structures during embryonic development. Linking virus-mediated changes in cellular pathways to embryonic origin of hearing loss needs precise molecular evaluation of the effect of each virus on a variety of tissues arising from the otic vesicle in order to differentiate into functional and structural units of the inner ear. This approach becomes even more important when considering cell-specific responses to different types of viral infections. However, comparing non-specific molecular alterations that viruses inflict to the vast majority of cell types for facilitating their entry or replication functions paves the path to predict the impact of these intracellular pathogens on signaling pathways regulating the development of the inner ear and the auditory system. The most common viruses that cause congenital and acquired hearing loss are discussed hereafter in terms of their relevance to primary molecular determinants of auditory system development during embryogenesis.

### 4.1. Cytomegalovirus

Among DNA viruses, CMV, a member of the Herpesviridae family which possesses a double-stranded enveloped genome, is known as the most prevalent cause of congenial SNHL. Assessment of temporal bones both in guinea pig models and humans have proved that CMV is not only able to synthesize viral proteins within inner ear compartments like the organ of Corti, scala media, and vestibular membrane, but also can contribute to the inflammation and fibrosis of cochlear ganglion by boosting the activity of macrophage inflammatory protein [8,9].

Such phenotypic observations have not been explained by obvious molecular mechanisms thus far. Wu et al. have demonstrated that CMV major immediate early protein 1 diminishes the expression of *SOX2* in neural progenitor cells through nuclear accumulation of unphosphorylated *STAT3* [64]. In addition to its function in maintaining the pluripotency of embryonic stem cells and neural development, *SOX2* is a key transcription factor that controls the axial specification of the otocyst and formation of the cochlear duct. Lack of *SOX2* during the development of the inner ear is associated with neural and sensory abnormalities in mice models [27]. Similarly, *SIX1* and *NGN1*, which are transcription factors with roles in axial specification of otic vesicle and neural fate determination in vestibulocochlear ganglion, were significantly downregulated by CMV infection in human fibroblasts and differentiating murine neural stem cells [65,66]. Moreover, these CMV-infected neural stem cells exhibit reduced expression of *WNT1*, a regulator in WNT pathway that directs the otic dorsal–ventral patterning, in addition to its fundamental function in the generation of the midbrain and cerebellum [66,67]. *WNT1* mutation results in defective and cystic inner ear structures in murine models [46], suggesting that CMV-related malformations in the inner ear might originate from viral protein interactions with WNT pathway components and regulators. *PAX2*, which plays crucial roles in the development and organization of midbrain and hindbrain, is another transcription factor that could be targeted by CMV. Browne and colleagues reported the downregulation of *PAX2* in CMV-infected primary fibroblasts at 1 and 48 h post infection [65]. Given that *PAX2* expression within the epithelium of otocyst modulates hindbrain-derived signals to begin the otic medial–lateral specification toward the generation of neuroblasts, CMV-induced *PAX2* downregulation could hypothetically disrupt this type of patterning in the otic vesicle, which might subsequently affect the segregation and migration of vestibular and auditory neuroblasts from the neural sensory domain into vestibulocochlear ganglion. Moreover, transcriptomic analysis of CMV-infected monocytes has shown that this virus can upregulate *JAG1*, a ligand that interacts with the receptors of Notch signaling pathway and is involved in sensory cell fate determination in the inner ear as well as formation of the organ of Corti [68,69]. Whether such a virus-mediated increase in the expression of *JAG1* could interfere with the natural development of hair cells and the organ of Corti needs further investigations in more physiologically relevant models (e.g., developing otocyst). Another possibility through which CMV might cause hearing impairment during embryogenesis is the reduced cellular level of *CDKN1B*, an enzyme inhibitor that regulates the cell cycle at G1 phase. Infection of lung and foreskin fibroblast cells with CMV contributes to the degradation of *CDKN1B* [65,70]. Interestingly, deletion of *CDKN1B* alters the development of cochlea through overproduction of hair cells and supporting cells, which subsequently lead to hearing loss in vivo [28]. Furthermore, during the development of the cochlear duct, *FGFR3* is known to be essential for some cochlear progenitor cells in order to differentiate into supporting cell variants, such as pillar cells, outer hair cells, and Deiters’ cells [59,60]. Absence of *FGFR3* could result in abnormal development of pillar cell [62]. It has been demonstrated that CMV major immediate early protein 1 interacts with *FGFR3* in human glioblastoma astrocytoma cell lines [71]. This interaction in CMV-infected human fibroblasts was noticed to increase the expression of *FGFR3* [72]. Because *FGFR3* also hampers the transformation of pillar cells into hair cells [73,74], its virus-evoked overproduction might change the natural balance between sensory and non-sensory cell fate specifications. Most of these discussed CMV-mediated molecular changes have not been validated in relevant models simulating the differentiation of the otic placode toward components of the inner ear and auditory system.

Treatment of CMV-infected neonates with Ganciclovir was found to halt SNHL development, despite causing neutropenia in some cases [75]. Due to in vivo evidence on embryotoxicity, Ganciclovir might not be administrated to pregnant women while infected with CMV. Furthermore, Valganciclovir has been reported to improve hearing outcomes in symptomatic infants with congenital CMV infection [76]. However, the efficacy of Valganciclovir depends on the severity of hearing abnormalities [77]. Hearing aids and cochlear implantation are usually recommended in cases where antivirals do not improve CMV-triggered SNHL.

### 4.2. Herpes Simplex Virus

HSV types 1 and 2 are double-stranded and enveloped DNA viruses that cause unilateral or bilateral SNHL in neonates. These viruses latently infect nerve cells innervating the primarily targeted tissues, which can later result in virus reactivation and disease recurrence. HSV-1 is more linked to hearing loss and encephalitis than HSV-2, even though hearing impairments were noticed to occur infrequently following HSV-1 infection except for cases involving severe neurological complications [78]. Reactivation of HSV after congenital or acquired infection often contributes to severe bilateral SNHL and might be associated with meningitis or encephalitis [79,80,81]. Based on in vivo evidences, both HSV-1 and HSV-2 infections lead to vestibular fibrosis, apoptosis of hair cells, and atrophy within intracochlear structures, such as tectorial membrane and stria vascularis [16]. Moreover, HSV viral capsids were detected in afferent and efferent endings of nerve fibers innervating the cochlea [11].

Despite the damage that HSV can do to the inner ear tissues, little is known about the molecular mechanisms by which this virus affects the auditory system, especially its developmental processes during early stages of embryogenesis. Similar to CMV, the expression of *SOX2* is significantly decreased in HSV-infected murine neural stem cells [82]. Such a deficiency in *SOX2* is consistent with HSV-related pathologies in cochlea that were noticed in animal models, as this transcription factor plays an important role in shaping the cochlear duct. Additionally, transcriptomic analyses of HSV-1-infected human fibroblasts indicated this virus remarkably upregulates the expression of *TBX1* [83], which is another transcription factor that not only interacts with retinoic acid signaling to control anterior–posterior patterning of the otocyst, but also participates in cochlear duct development and coiling [28,31]. Whether overexpression of *TBX1* leads to disruption of these developmental events within the inner ear is an unanswered question. The same transcriptomic data revealed that HSV-1 increases the expression of *SOX3* and *GLI3* [83]. Like *TBX1*, *SOX3* is also involved in regulating anterior–posterior specification of the otocyst via interaction with retinoic acid signaling [32]. *GLI3* is the activator of the SHh pathway and needs to be activated for the formation of the distal part of the cochlear duct from ventral region of otocyst. However, due to the inhibitory function of *GLI3*, the creation of proximal part of the cochlear duct and structures like the saccule and utricle requires lower levels of the repressor molecule to regulate SHh activity [34]. It is not known if this HSV-induced dysregulation of *GLI3* in physiologically relevant models could affect any intracochlear components. JAG1, which is upregulated in CMV-infected monocytes, also seems to be overexpressed in human fibroblasts [68,83]. As discussed earlier, depending on targeted cell type and stage of development, this virus-mediated alteration of *JAG1* by either CMV or HSV might influence the formation of sensory hair cells and the organ of Corti. Unlike CMV that degrades *CDKN1B* in fibroblast cells, Sánchez-Quiles and coworkers reported that HSV-1 infection elevates the expression of *CDKN1B* in human hepatoma cells [84]. Increased levels of *CDKN1B* might reduce the production of hair cells and supporting cells but it is not clear if the upregulation of this molecule could lead to hearing loss like its deletion by CMV. None of these mentioned molecular predictions have yet been examined in appropriate cellular or animal models.

Some clinical trials have demonstrated that antiherpetic drugs like famciclovir can reduce abnormal auditory fluctuations in adults whose HSV infections developed into Meniere’s disease, but may not improve vertigo and tinnitus [85]. Depending on other HSV-associated outcomes such as encephalitis and meningitis, different doses of Acyclovir were found to ameliorate some cases of hearing loss that occurred after infection with this virus [11].

### 4.3. Rubella Virus

RV is a non-enveloped pathogen that until 2018, belonged to the virus family Togaviridae. It has since been classified as a member of the new family Matonaviridae. These viruses have a single-stranded RNA genome. As a teratogen, this virus is known to result in bilateral SNHL in almost 60% of congenial infections occurring within the first trimester of pregnancy [12]. RV can directly affect cochlea by inducing apoptosis in stria vascularis of the cochlear duct and the organ of Corti [10]. The strial tissues can be infected with RV as well, which might eventually alter the structure of the endolymph [12].

The molecular mechanisms of RV-mediated hearing loss at either neonatal or fetal stages have remained elusive so far. In contrast to CMV, which downregulates *SIX1* in neural stem cells and fibroblasts, Geyer et al. reported the upregulation of *SIX1* in RV-infected adult endothelial cells [86]. As mentioned earlier, *SIX1* is a transcription factor that regulates otic vesicle patterning and neural fate specification in the vestibulocochlear ganglion. *SIX1* deletion was seen to cause neural and sensory defects in mice [25,87]. Nevertheless, the overexpression of *SIX1* is mostly associated with malignancies in various tissues but has not yet been directly related to abnormalities involving vestibulocochlear ganglion and otic vesicle specification. Regardless of *SIX1* and other main molecular determinates of inner ear development, gene expression profiling of RV-infected primary fetal endothelial cells showed the significant downregulation of a few genes that indirectly control the formation of inner ear and auditory system, such as *FZD3*, *JAG2*, *NOG*, and *SLC25A27* [86]. Interestingly, RV infection of pluripotent stem cells differentiating toward endoderm, which is assumed to be a relevant in vitro model for mimicking the initial development of the auditory system and differentiation into the epithelium of the auditory tube, did not indicate RV-associated molecular alterations corresponding to inner ear development [88]. Given that RV is capable of inducing fundamental changes in some developmental networks like TGF-β and Wnt signaling pathways, further attempts using relevant cell types are required to uncover whether RV can affect the main molecular determinants that give clues to embryonic stages of auditory system development.

Because of global vaccination programs, RV is assumed to be an eradicated infectious disease in several countries. Nevertheless, this pathogen is still circulating across the world through areas that do not properly pursue immunization measures [89]. No antiviral therapy is effective in neonates, either to suppress RV or to reduce hearing complications.

### 4.4. Lymphocytic Choriomeningitis Virus

Lymphocytic choriomeningitis virus (LCMV) belongs to the family Arenaviridae and is a rodent-borne enveloped virus that contains a single-stranded RNA genome. Irrespective of spontaneous abortion, infection with this teratogenic virus during the first and second trimesters can cause severe to profound SNHL in offspring [11]. No information is available to explain how LCMV can affect a developing structure within the embryonic or fetal auditory systems.

Isolation of CD8^+^ T cells from LCMV-infected mice has confirmed the upregulation of *GLI2*, which is required under the same expression pattern for the development of the cochlear duct [33,90]. However, the pathogen-induced overexpression of *GLI2* in T lymphocytes might not necessarily correspond to the same LCMV infection of a developing otocyst. LCMV could also potentially target crucial signals controlling otic patterning and migration of auditory neuroblasts. For instance, Parmigiani and colleagues have indicated that postnatal LCMV infection decreases the expression of *PAX2* in murine astroglial-like neurogenic progenitors residing in the prospective white matter of the cerebellum [91]. As described earlier, CMV infection can similarly downregulate *PAX2* in fibroblasts [65]. *PAX2* is necessary for medial–lateral specification of otic vesicle to delaminate neuroblasts, suggesting that LCMV can probably interfere with the generation or migration of vestibular and auditory neuroblasts toward vestibulocochlear ganglion. Likewise, LCMV infection was shown to overexpress *CDKN1B* while reducing the expression of *LMX1A* in CD8^+^ effector T cells [90]. Because *LMX1A* is highly expressed in auditory neuroblasts, virus-mediated downregulation of this transcription factor implies that LCMV could negatively affect the formation and proliferation of neuroblasts during the development of the otocyst. Moreover, restricting the function and expression of *LMX1A* in animal models disrupts the generation of different compartments within the inner ear, including the non-sensory structure, the endolymphatic duct, and the membranous labyrinth [40]. Like HSV-1-infected hepatoma cells, the upregulation of *CDKN1B* in CD8^+^ effector T cells following LCMV infection might not result in hearing impairments if it occurs during the specification of the otic vesicle, but possibly could decrease the proliferation of hair cells and supporting cells. *ATOH1* is another molecule through which LCMV can influence hair cells. LCMV infection in neonatal rat models elevates *ATOH1* mRNA levels in the cerebellum [92]. Since *ATOH1* is involved in the generation of sensory hair cells, LCMV infection during the development of the cochlear duct has the capacity to alter the normal production of these cells. In addition to sensory hair cells, LCMV might be able to hinder the differentiation of cochlear progenitor cells into various types of supporting cells. Such a dysfunction could be triggered by LCMV-induced downregulation of *FGFR3*, a situation that has been demonstrated to happen in murine hepatocytes after LCMV infection [93]. Moreover, as mentioned before, *FGFR3* plays an important role in balancing sensory cell fate specification. In contrast to CMV, which was found to directly overexpress *FGFR3* in human glioblastoma astrocytoma cell lines, the *FGFR3* downregulation by LCMV highlights the possibility that this virus not only could affect the formation of supporting cells but also could disturb the normal mechanism controlling sensory and non-sensory cell fates. Because most reported conditions have been tested in irrelevant models, further evaluations are needed to determine how differently a developing otic vesicle would respond to LCMV infection.

Ribavirin, which inhibits viral RNA synthesis, has been widely used to treat LCMV infection in adults, despite low efficacy and side effects like hemolytic anemia [11]. Moreover, teratogenicity of Ribavirin in animal studies suggest that this antiviral should not be administrated during pregnancy [94]. No antiviral agent has been found to remediate LCMV-induced hearing impairment in neonates so far. Hearing aids are recommended for treating SNHL in newborns with congenital LCMV infection.

### 4.5. Human Immunodeficiency Virus

As a retrovirus, HIV contains a single-stranded RNA genome that transforms into a double-stranded DNA after hijacking the host cell transcription machinery. Despite the potency for affecting a variety of cell types, HIV often tends to infect immune cells and neurons. HIV infection causes both congenital and acquired hearing loss. Most in utero exposure cases of HIV infection result in CHL in neonates, while infection among adults can contribute to mild to moderate levels of SNHL at high frequencies [15,95,96,97]. However, it is often difficult to directly link HIV to the induction of CHL. A set of studies showed that HIV targets both the central and the peripheral auditory systems. Apart from detection of HIV components in auditory and vestibular hair cells, tectorial membrane, and strial cells, abnormalities in ABRs in HIV-infected subjects suggest that this virus might affect the auditory nerve [13,14,15].

As with other viruses, no precise molecular data is available on HIV-induced hearing loss with embryonic origin. Exposure of neural progenitor cells to HIV-1 Tat protein can decrease the expression of *SOX2* [98]. As discussed, the same impact on *SOX2* expression was also noted after infection of similar cells with CMV and HSV, indicating that *SOX2*-related auditory development processes like axial specification of the otocyst and generation of the cochlear duct also can be affected by HIV. Furthermore, transfecting HIV-1 Tat protein into Jurkat cells elevates the expression of *LFNG* [99]. This transferase regulates the formation of the cochlear duct and the cell fate specification from neural sensory competent domain. Nevertheless, deletion of *LFNG* in animal models has not been associated with obvious malformations or defects within the inner ear structures [100]. It is unclear whether HIV-induced upregulation of *LFNG* in the otocyst could result in abnormalities in cochlea or sensory cell fate specification. According to single cell analyses, HIV also downregulates *SOX3*, *RBPJ*, and *POU3F4* in primary CD4+ T cells [101]. *SOX3* interacts with other molecules in retinoic acid signaling to regulate anterior–posterior specification of the otocyst [32]. Thus, its reduced expression following HIV infection could restrict the normal development of the inner ear, mainly due to the involvement of this axis specification in creating both sensory and non-sensory tissues of the auditory system. Furthermore, *RBPJ* is a Notch signaling pathway regulator that plays a fundamental role in sensory cell fate determination during the development of the otic vesicle [44,45]. Because lack of *RBPJ* leads to sensory defects, it is possible that HIV infection would have the same effect on the structures derived from the developing otocyst. *POU3F4* downregulation by HIV might influence the outgrowth and coiling of the cochlear duct, as the absence of this molecule was demonstrated to cause defective coiling and shrinkage of the cochlear duct [53]. Validation of these molecular predictions in models simulating the otic vesicle specification could uncover the HIV capacity for interfering with the initial stages of auditory system development.

Highly active antiretroviral therapy elevates the number of CD4+ T cells, eventually restricting opportunistic infections that cause hearing loss in HIV-infected individuals [102]. However, such a therapy does not significantly recover SNHL in infected neonates. Infected patients with mild to moderate SNHL are often using hearing aids, while cochlear implantation is a better course for those who experience severe to profound hearing loss [11,103].

### 4.6. Viruses Causing Acquired Hearing Loss

Although viruses causing acquired hearing loss are affecting people postnatally, the possible transplacental passage of these viruses within the initial stages of embryogenesis might restrain the normal development of the auditory system and the inner ear compartments. These viruses include measles (MV), mumps, and West Nile virus (WNV), which all contain a single-stranded RNA genome. Varicella zoster virus (VZV), another herpesvirus, is a different example of viral pathogens contributing to acquired hearing loss as it encompasses a double-stranded enveloped DNA.

Even with widespread vaccination, bilateral profound SNHL can still be seen following MV infection among those inhabiting certain areas where immunization programs are not properly followed [104,105]. Analyses of temporal bones in both human and in vivo models have reported not only cellular infiltration within cochlea but also degeneration and abnormalities in cochlear neurons, the organ of Corti, and stria vascularis [11]. Moreover, due to the temporary inactivation of immune responses, MV infection leads to the inflammation of the middle ear (Otitis media) in a significant number of infected people [106]. Based on the detection of viral antigens and histological assessments, MV is assumed to cause otosclerosis, a condition in which the bone of middle and the inner ear grows abnormally, ultimately resulting in SNHL and CHL [107]. However, none of these observations appear to be related to molecular determinants of auditory system development at early stages of embryonic growth, except for the transcription factor *PAX2*. Infection of human dendritic cells with MV leads to *PAX2* upregulation [108]. The opposite expression pattern was found for this molecule following infection with CMV and LCMV in other cell types, such as fibroblasts and astroglial-like progenitors. As discussed earlier, *PAX2* guides hindbrain signals to provoke medial–lateral specification of the otic vesicle, which then helps to form auditory neuroblasts. MV-induced upregulation of *PAX2* might dysregulate neuroblast generation if occurs during such a crucial patterning stage of the otocyst. Regardless of hearing aids and cochlear implantation, no antiviral has been reported to improve hearing dysfunctions in MV-infected children [105].

Acquired SNHL after Mumps infection is usually reversible and unilateral [11]. Studies have isolated Mumps antigens from endolymph and perilymph [109]. Various hypotheses have been put forward regarding the causes of SNHL following Mumps infection, most notably including but not limited to stria vascularis, damaged myelin sheath covering vestibulocochlear nerve, and atrophy of hair cells within the organ of Corti [11,105,109]. These Mumps-oriented assumptions are not directly linked to molecular hallmarks of otic vesicle development. Further research is needed to reveal the molecular aspects of these potential abnormalities during embryogenesis, fetal growth, and postnatal stages. Similar to MV and RV, treatment of mumps-related SNHL does not include any specific antiviral agent and mainly involves hearing aids and cochlear implantation, depending on the severity of hearing loss [110,111].

People who become infected with WNV often do not show neurological complications and might remain asymptomatic [11,112]. WNV-associated SNHL seems to occur rarely and could emerge mostly in immunocompromised individuals [113,114]. Despite the capability of this virus for congenial transmission, nothing is known about the molecular mechanisms by which WNV might lead to hearing loss either at early pregnancy or after birth. Among molecules regulating the initial processes of inner ear development, transcription factors *SOX2* and *GBX2* were reported to be upregulated by WNV in horse brain tissues [115]. In contrast to WNV, as mentioned above, a few other viruses like CMV, HSV-1, and HIV tend to decrease the expression of *SOX2*. Considering the roles of these molecules in the patterning of otocyst and formation of cochlea, more studies in relevant models are needed to determine whether WNV-mediated overexpression of *SOX2* and *GBX2* could negatively affect otic vesicle development. Recovery from hearing loss after WNV infection occurs spontaneously in most cases [113,114]. Some WNV-associated severe neurological complications were mitigated by immunoglobulin, Ribavirin, and IFN-α2b treatment [112].

The latency and reactivation capacities of VZV in neurons are considered possible mechanisms through which this virus leads to SNHL. Following the reactivation of VZV in geniculate ganglion and inflammation, the Vestibulocochlear nerve turn into a bridge to transfer this virus into the auditory canal [11]. Clinical reports confirmed that VZV-associated hearing loss can result in cochlear nerve bleeding, degeneration of the organ of Corti, death of geniculate ganglion cells, more infiltrating lymphocytes, demyelination, and axonal loss [116]. Transcriptomic assessment of VZV-infected sensory ganglia has shown the reduced expression of *SOX2* and *PRX* [117]. Because PRX is involved in the formation of semicircular canals, it is possible that VZV could potentially target the balance and sensory input for rotary movements by decreasing the expression of *PRX* in the otocyst, in addition to disrupting cochlea development by *SOX2* downregulation. Antiherpetic drugs and corticosteroids like acyclovir and prednisone are usually prescribed for recovering VZV-related SNHL cases [11].

### 4.7. Other Viruses that Might Potentially Affect Inner Ear Development

Zika virus (ZIKV) has been recently shown to contribute to hearing loss in children who survived in utero infection [118]. Our recent proteomic analyses of ZIKV infection in Vero cells detected dysregulations in the expression of several proteins involved in hearing loss [119]. Interestingly, we identified the downregulation of *FGFR3* in our datasets, which is one of the main molecular determinants of otocyst development and generation of supporting cells. Yan and colleagues demonstrated the reduced expression of *BMP4* following ZIKV infection of neural crest cells [120]. Such a downregulation of *BMP4* suggests ZIKV potential for targeting the structures within the cochlear duct. Infection of human neural stem cells with ZIKV was noted to elevate the expression of *CDKN1B* [121], a condition that might lead to hearing loss through overproduction of hair cells and supporting cells. Genomic studies indicate that *HMX3*, the homeobox transcription factor that interacts with FGF signaling pathway, can be downregulated by ZIKV in neural progenitor cells [122]. This downregulation might affect the normal formation of semicircular canals, which influences the vestibular system. Injecting ZIKV into the neural tube of chicken embryos reduced the expression of *FGF8* in the posterior isthmus [123]. *FGF8* downregulation by ZIKV in physiologically relevant model for otic vesicle development could test the hypothesis that this virus may hamper the generation of supporting cells.

Infection with influenza A virus (IAV) has not been reported to cause severe types of persistent hearing loss. Nevertheless, in vitro studies demonstrated the differential expression of a few molecules that are among the key regulators of inner ear development. For instance, infection of human induced pluripotent stem cells with IAV diminishes the protein production of *SOX2* [124]. Terrier et al. found that both H1N1 and H3N2 subtypes of IAV can decrease gene expression of *LFNG* in human A549 lung epithelial cell line [125]. We have already identified the downregulation of *JAG1* protein after H1N1 infection in primary human bronchial airway epithelial (HBAE) cells [126]. Although lacking strong evidence on the possibility of targeting a developing embryo, molecular alterations of *SOX2*, *LFNG*, and *JAG1* suggest that IAV is hypothetically capable of interfering with several key processes controlling auditory system development, including otic vesicle patterning, neural fate determination, cochlear duct formation, and generation of sensory hair cells.

During the recent COVID-19 pandemic, a few cases of SNHL have been documented following exposure to coronavirus [127,128,129]. Irrespective of the vague cellular and molecular etiology, screening the latest omics studies involving coronavirus pathogenesis could provide valuable information for matching both markers of postnatal hearing loss and the main genes involved in the primary stages of auditory system development with virus-induced changes in host. For example, analyzing lung transcriptomic data showed the reduced expression of *FGFR3* [130], one of the regulators for generating supporting cells. Moreover, another transcriptomic profiling of primary human lung epithelium after coronavirus infection reported the downregulation of *HEY2* [131]. This reduced expression of *HEY2* could influence supporting cells if expressed in the same direction in physiologically relevant tissues because both *FGFR3* and *HEY2* play a common role in conducting signals from hair cells to make different supporting cells.

## 5. Concluding Remarks

**Figure 1:**
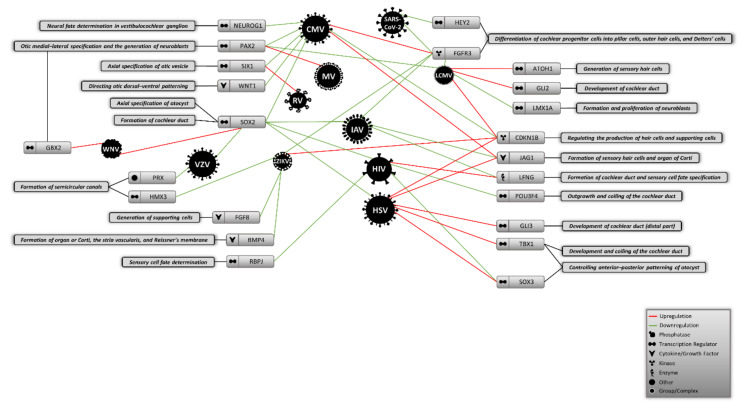
Proposed model of potential virus-induced molecular alterations during the initial stages of auditory system development. CMV: Cytomegalovirus, MV: Measles virus, LCMV: Lymphocytic choriomeningitis virus, RV: Rubella virus, IAV: Influenza A virus, HIV: Human immunodeficiency virus, HSV: Herpes simplex virus, ZIKV: Zika virus, VZV: Varicella zoster virus, WNV: West Nile virus.

The embryonic origins of virus-induced hearing loss have been neglected in most studies to date. Investigating the earliest stage of development gives a broad insight into the mechanisms that different teratogenic and congenital viruses might utilize to cause abnormalities during the development of inner ear and compartments of auditory system. This approach could also help to explain the missing parts of the molecular etiologies of virus-mediated hearing loss in neonates. In this review, in addition to discussing the main cellular and molecular hallmarks of inner ear development during embryogenesis, we performed a deep literature molecular screening to determine whether viruses causing congenital and acquired hearing loss could alter the expression of any molecules that control the initial stages of auditory system development. As shown in Figure 1, numerous viruses were found to differently change the expression of some key molecular factors involved in the formation of inner ear structures. We found that molecules like *SOX2*, *FGFR3*, and *CDKN1B* could be more affected by different viruses than other molecules shaping the auditory system, suggesting that cochlear development and production of hair and supporting cells might be disrupted by certain viral infections during embryogenesis. Given that this information is sorted based on different cell types that might not actually be linked to the auditory system, further validations are required to using appropriate in vitro models that mimic the differentiation and specification of this system from the otic placode.
